# Poly(N-vinylcaprolactam) Nanogels with Antiviral Behavior against HIV-1 Infection

**DOI:** 10.1038/s41598-019-42150-9

**Published:** 2019-04-05

**Authors:** Micaela A. Macchione, Carlos Guerrero-Beltrán, Anabella P. Rosso, Esteban M. Euti, Marisa Martinelli, Miriam C. Strumia, Maria Ángeles Muñoz-Fernández

**Affiliations:** 1 Universidad Nacional de Córdoba, Facultad de Ciencias Químicas, Departamento de Química Orgánica. Av. Haya de la Torre esq. Av. Medina Allende, Córdoba, X5000HUA Argentina; 2 CONICET, Instituto de Investigación y Desarrollo en Ingeniería de Procesos y Química Aplicada (IPQA). Av. Velez Sárfield 1611, Córdoba, X5000HUA Argentina; 3 Sección Inmunología, Laboratorio Inmuno Biología Molecular, Hospital General Universitario Gregorio Marañón, Madrid, 28007 Spain; 4 Instituto de Investigación Sanitaria Gregorio Marañón (IiSGM), Madrid, 28007 Spain; 5 Spanish HIV HGM Biobank, Madrid, 28007 Spain; 6 Networking Research Center on Bioengineering, Biomaterials and Nanomedicine (CIBER-BBN), Madrid, 28029 Spain

## Abstract

Stimuli-responsive nanogels offer promising perspectives for the development of next generation formulations for biomedical applications. In this work, poly(N-vinylcaprolactam) nanogels were synthesized varying the concentration of monomer and crosslinking agent. Thus, the inhibitory effect of poly(N-vinylcaprolactam) nanogels against HIV-1 infection is presented for the first time. In particular, we have demonstrated that one of the synthesized poly(N-vinylcaprolactam) nanogels with initial concentration of 80 mg of vinylcaprolactam and 4% of crosslinking agent shows antiviral behavior against HIV-1 infection since this nanogel inhibits the viral replication in TZM.bl target cells.

## Introduction

The Joint United Nations Program on HIV/AIDS (UNAIDS) estimates that approximately 37 million people were living with HIV-1 worldwide at the end of 2016^1,2^. In addition, HIV-1 sexual transmission is held accountable for around 80% of all infections, with roughly half of the affected individuals being women^3^. In the last years, the use of long-lasting, female-controlled and efficacious topical microbicides to decrease and control HIV-1 epidemic among women has strongly emerged^4–7^.

Many compounds with anti-HIV-1 activity have been tested as topical microbicides, such as sulfated polysaccharides like carrageenan and dextran sulfated^8,9^, drugs like tenofovir^10–12^ or dapivirine^13^, and nanostructures like dendrimers^14–16^. However, most of them have failed in the clinical trials due to vaginal toxicity and irritation, increasing the susceptibility to sexually transmitted infections rather than providing protection^17,18^. Recently, a patent in which hypotonic formulations of synthetic hydrogel based on poloxamer were used as vaginal or colorectum mucosal barriers was published^19^. Its ability for trapping viruses including HIV was demonstrated. This work constitutes, to the best of our knowledge, the only report of the use of synthetic polymeric hydrogels as anti-HIV agents by themselves. However, the development of polymeric formulations with low polydispersity values and a well-defined molecular weight results challenging. In this sense, nanogels (NGs) have emerged as sized-controlled nanostructures with important biomedical applications. NGs are three-dimensional-crosslinked polymeric matrices that have nanometric sizes in their three dimensions^20^. NGs combine the solids and fluids properties, and some of their properties depend on the contributions of each portion^21^. The liquid presence prevents the network from collapsing into an insoluble compact mass and the solid part prevents the liquid from flowing freely.

NGs have unique properties, such as, high encapsulation efficiency and protection of active agents from degradation, which make them ideal candidates as drug delivery or theranostic systems. In addition, the hydrophilic nature of NGs gives high biocompatibility, which represents a clear advantage over other types of nanomaterials in this kind of applications^22^. In other words, unique multifunctionality and stability of NGs are difficult to find in other types of nanosystems^23,24^. For example, inorganic nanomaterials such as quantum dots and magnetic and gold nanoparticles, that have numerous applications, including against sexual infection diseases (SIDs), have low colloidal stability and are rapidly eliminated by the mononuclear phagocytic system^22^.

Moreover, stimuli-responsive NGs respond to external stimuli (pH, temperature, magnetic field, redox environments, among others)^25^. These smart materials offer new promising perspectives for the development of next generation therapeutic agents. Considering all the stimuli-responsive systems, poly(N-vinylcaprolactam) (PVCL) is one of the most studied thermo-responsive polymers^26–30^, after poly(N-isopropylacrylamide) (PNIPAm)^26^. Recent studies have reported that PVCL is biocompatible, and then it is very attractive for biomedical and environmental applications^26^. Also, the possible hydrolysis of amide group of VCL will produce a polymeric carboxylic acid, which is innocuous for biomedical applications. On the contrary, PNIPAm can yieldsmall toxic amide compound^31^. However, deeper studies on the synthesis, functionalization, characterization and applications of PVCL-based NGs are required.

PVCL displays a thermo-responsive behavior, with a characteristic lower critical solution temperature (LCST). Accordingly, below LCST the polymer swells in water and after heating, it shrinks rapidly to a collapsed state^32^. It has been found that for PVCL hydrogels the swelling-collapse behavior is completely reversible with the same LCST for both processes, between 30 and 32 °C^26,33^.

NGs have not been tested previously as antiviral agent for inhibition of HIV-1 infection. In this context, we present, for the first time, the effect of NGs against the HIV-1 infection. Particularly, we have demonstrated that a PVCL NG with initial concentration of 80 mg of VCL and 4% of crosslinking agent (PVCL80_4_ NG) can be considered as new potential microbicide against the HIV-1 infection since the PVCL80_4_ NG inhibits the viral replication in TZM.bl target cells.

## Experimental Section

### Reagents

N-Vinylcaprolactam (VCL) was obtained from Aldrich; N,N′-methylenebisacrylamide (BIS) (99%) from Sigma-Aldrich; N,N,N′,N′ tetramethylethylenediamine (TEMED) (98%) from Fluka; potassium persulfate (KPS) from Merck; sodium dodecyl sulfate (SDS) (96%) from BioPack; 3-(4,5-dimethylthiazol-2-yl)-2,5-diphenyltetrazolium bromide (MTT) from Sigma-Aldrich; dimethyl sulfoxide (DMSO) from Sigma-Aldrich; nuclease-free water from Promega, Madrid, Spain; phosphate-buffered saline (PBS) from Lonza, Walkersville, MD, USA; Dulbecco’s Modified Eagle’s Medium (DMEM) from Biochrom, AG, Germany; supplemented with 5% heat-inactivated-fetal bovine serum from Life Technologies, Madrid, Spain and a cocktail of antibiotics. Dialysis membranes of cellulose ester with a molecular weight cut-off of 50 kDa were purchased from SpectrumLabs. All chemicals were used as purchased. MilliQ grade water was employed as the polymerization medium.

### Synthesis of nanogels

PVCL NGs were prepared by thermo-precipitation in aqueous phase via free radical polymerization as follows: appropriate amounts of VCL and crosslinker (BIS) and, 0.0013 g of SDS (between 0.8–1.0%), were added to 3 mL MilliQ grade water. The solution was purged with nitrogen for 15 min under continuous stirring to remove dissolved oxygen. The reaction was carried out at 70 °C for about 5 h after the addition of 1% mol of KPS and 1% mol TEMED to initiate the polymerization by thermal decomposition. After the reaction, the NGs were purified by dialysis with membranes of cellulose ester with a molecular weight cut-off of 50 kDa against distilled water (2 liters) for one week (1 change of water per day) to remove unreacted monomers and the surfactant. Subsequently, for biological assays the samples were freeze-dryed obtaining a white solid.

The following NGs were obtained: PVCL80_0_, PVCL80_1_, PVCL80_2_, PVCL80_4_, PVCL60_0_, PVCL60_4_ and PVCL60_10_. They were called according to their composition as PVCL**x**_**y**_, where ***x*** means mg of VCL and ***y*** mol percentage of BIS respect to mols of VCL.

### Spectroscopic analysis

Fourier transform infrared spectra (FT-IR) bands of the lyophilized NGs were obtained on a Nicolet iN10 (Thermo Scientific, USA; ST 2425-CONICET) using a liquid nitrogen cooled detector of Mercury Cadmium Tellurium (MCT). Data were analyzed using the OMNIC 8.3 Picta software package.

Nuclear magnetic resonance spectra were recorded on a Bruker Ultra Shield 400 (^1^H-NMR: 400 MHz ST 1027-CONICET) in deuterated water. Data were analyzed using the Mestre Nova software package.

### Average particle diameter and phase transition temperature

Average hydrodynamic particle diameter (D_h_) at different temperatures and the phase transition temperatures (Tp), were determined by dynamic light scattering (DLS) in a Nano Zetasizer instrument with a He–Ne laser (λ = 633 nm) and scattering angle of 173°. Measurements were made in a temperature range from 15 °C to 50 °C with a temperature equilibrium time of 120 s. All samples were prepared with a dilution of 1 in 10.

Cloud point temperatures (Tcp) were measured on a Shimadzu 1800 UV-vis spectrophotometer. Suspensions of NGs were heated from 15 to 50 °C while monitoring every 2 °C both the transmittance at 600 nm (1 cm path length) and the solution temperature. The Tcp of each NG was determined using the inflection point of the curve of the absorbance vs. temperature.

### Thermogravimetric Analysis (TGA)

Thermal degradation curves of NGs were performed on a Shimadzu DTG-60 (Japan) (ST 2901-CONICET), measurements were carried out from room temperature to 800 °C at a heating rate of 10 °C/min under a pure nitrogen atmosphere.

### Size and morphology of nanogels

Transmission electron microscopy (TEM) observations were carried out on a JEOL JEM EXII 1200 of high contrast, operating with 80 kV (INTI-IPAVE-CONICET). Samples were prepared on carbon-coated copper grids of 200 mesh from TED PELLA. The solution of each NG was dripped on a grid and left to dry at room temperature. Then, they were covered with a 2% uranyl acetate solution and again allowed to dry at room temperature.

The size distribution determined from TEM images was performed by using Image J software. Average diameter values were obtained from populations of 200 particles.

### Cell viability assays

PVCL NGs toxicity was determined on TZM.bl cell line by MTT assay according to the manufacturer’s instructions by using a Synergy 4 Plate Multileaver, Biotek Instrument (USA). A stock suspension of NGs (in order to give 100 µg/mL in the experiment) was prepared by weighing the white solid obtained after freeze-drying and mixing it with nuclease-free water. Then, this sample was sonicated by 15 min to obtain a stable suspension. Lower-concentration suspensions were prepared by dilution of this stock suspension using nuclease-free water. DMSO (10%) and culture medium were used as positive and negative controls of cellular death, respectively. Each experiment was performed in triplicate.

### Inhibition of HIV-1 replication experiments

Cells were seeded in p96-well plates 24 h prior to experiments (15 × 10^3^cells/well). TZM.bl cells were treated with non-toxic concentrations of PVCL NG for 1 h before infection with R5-HIV-1_NLAD8_ isolate (30 ng of p24/10^6^cells) under culture conditions. After 3 h, cells were washed twice with sterile phosphate-buffered saline (PBS) and incubated for 48 h at 37 °C and 5% CO_2_. Cells were lysed and the percentage of HIV-1 infection was determined by quantification of luciferase activity (Luciferase Assay System, Promega). G2-S16 dendrimer (10 µM) was used as a positive control of HIV-1 inhibition^34,35^. Each experiment was performed in triplicate.

## Results and Discussion

**Figure 1:**
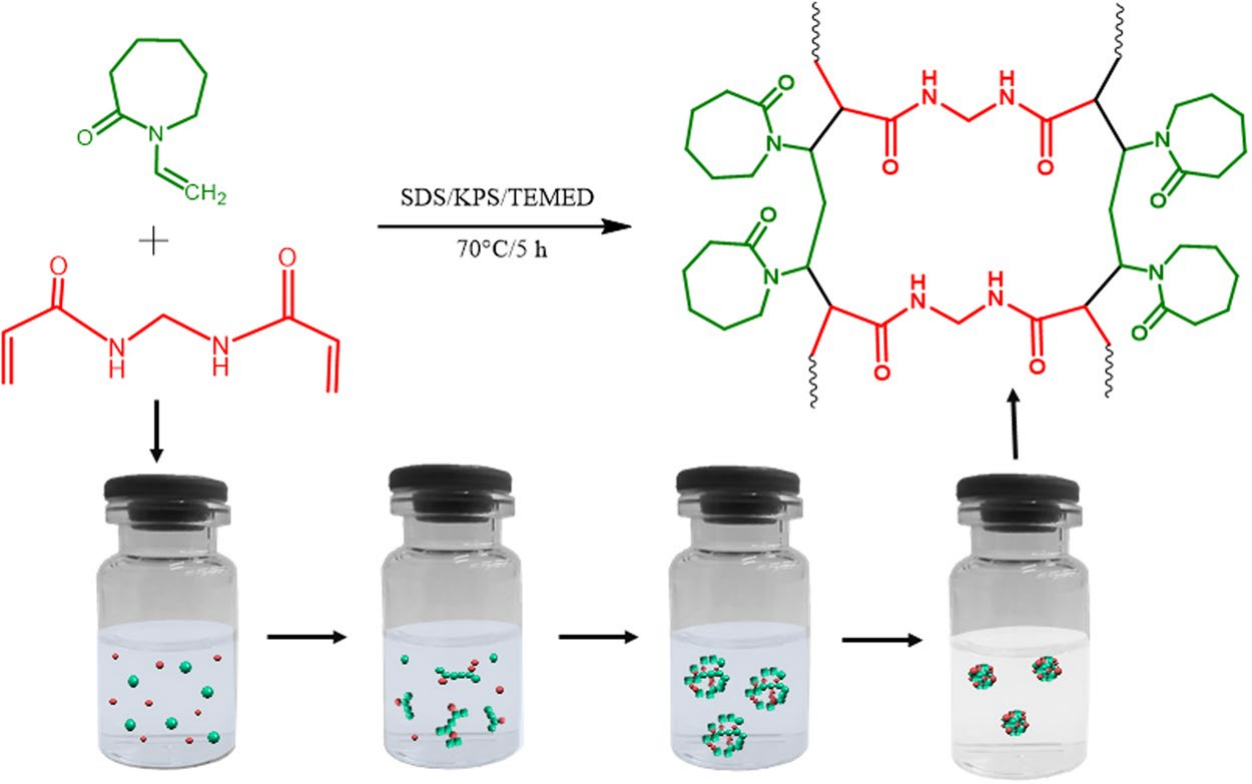
Series of NGs from PVCL prepared by thermo-precipitation in aqueous phase.

In this work, a series of NGs from VCL were prepared by thermo-precipitation in aqueous phase in the presence of a surfactant (SDS) to control the size and reduce the distribution of the particles (Fig. 1). The polymerization was carried out at 70 °C, a temperature above the LCST of PVCL, and the reaction begins with a thermal initiator (KPS). When the chain length of the oligomers grows, their solubility in water decreases and the previous homogeneous systems becomes heterogeneous with particles that form a dispersed phase. Within the dispersed phase, the particles grow as the polymerization continues until the so-called “critical length” in which the polymerization is stopped. The reaction was initiated via free radical polymerization by the thermo-initiator KPS/TEMED. The polymeric NGs were synthesized by varying monomer and crosslinker ratios. NGs were purified through dialysis against water and obtained as a white solid after freeze-drying.

### Spectroscopic analysis

**Figure 2:**
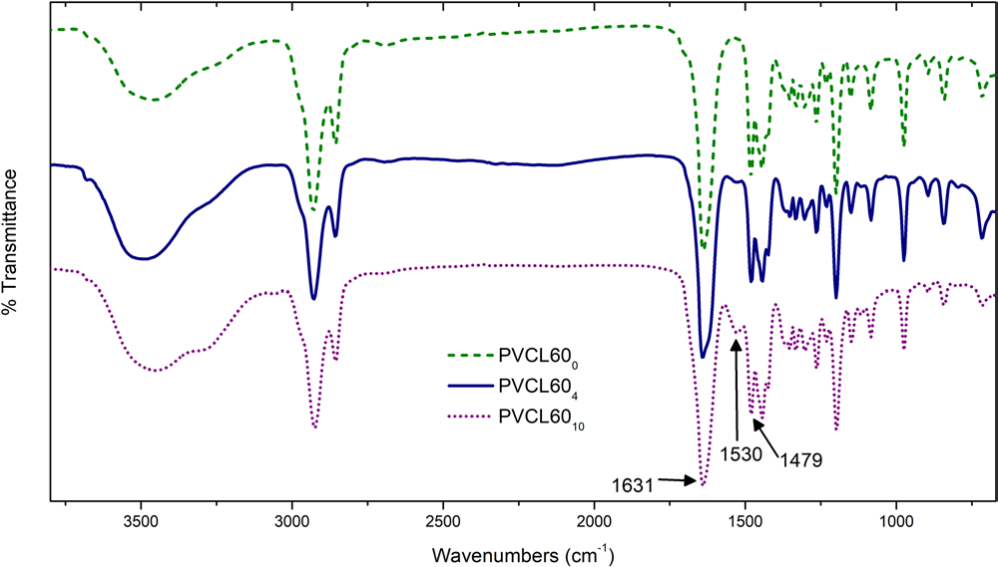
FT-IR spectra of PVCL NGs.

In order to confirm the polymerization of VCL and the crosslinking with BIS, FT-IR and ^1^H-NMR spectroscopy measurements were carried out. Figure 2 shows FT-IR spectra corresponding to VCL and NGs. The FT-IR spectrum of the PVCL (without crosslinker) exhibited the characteristics bands at 2900–3000, 1631, and 1479 cm^−1^ corresponding to the stretching vibration of the C-H, C=O, and C-N, respectively^36^.

**Figure 3:**
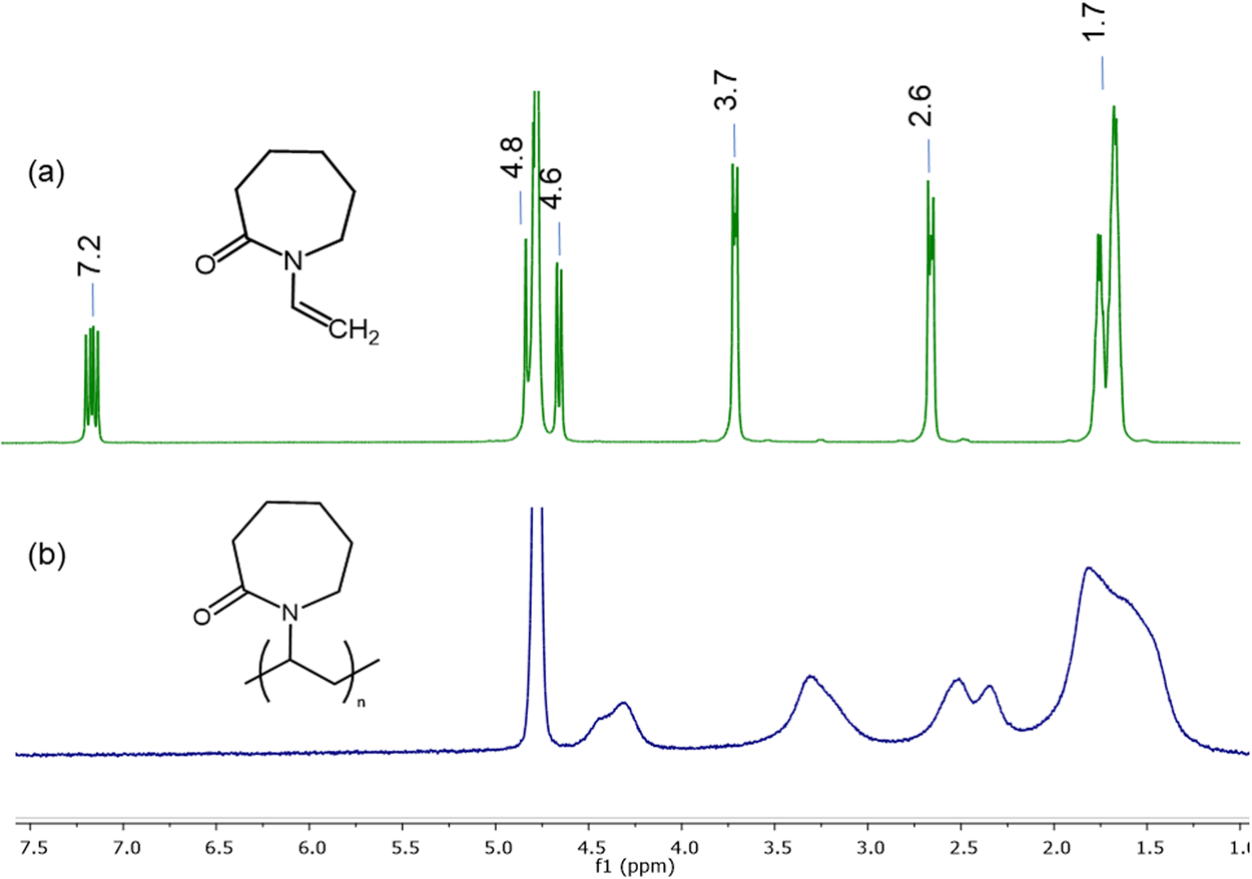
^1^H-NMR spectra of VCL monomer and PVCL NG.

On the other hand, the FT-IR spectrum of the crosslinked NGs with BIS also showed a signal at 1530 cm^−1^ corresponding to the deformation of the N-H (amide group) and it was clearly observed that the relative intensity of this signal increased with the amount of crosslinker. The ^1^H-NMR spectrum of the monomer VCL exhibited the peaks at 7.2, 4.8 and 4.6 ppm (1H each, =CH) corresponding to the vinyl protons, and the peaks at 3.7, 2.6 (2H each, –CH_2_) and 1.7 ppm (6H, −CH_2_) corresponding to the caprolactam ring^37^. Regarding at the ^1^H-NMR spectra of the PVCL NGs was possible to observe the typical widening of bands as well as the disappearance of the signals coming from the vinyl protons. These facts confirm the polymerization reaction and the absence of free monomer (Fig. 3).

### Average particle diameter and phase transition temperature

A large number of variables influence the nanostructure of the NGs and their thermo-responsive behavior, such as type of solvent, concentration of polymer, degree of crosslinking and ionization, among others^30^. In order to evaluate structure and thermo-responsive behavior, a series of NGs were synthesized.

**Figure 4:**
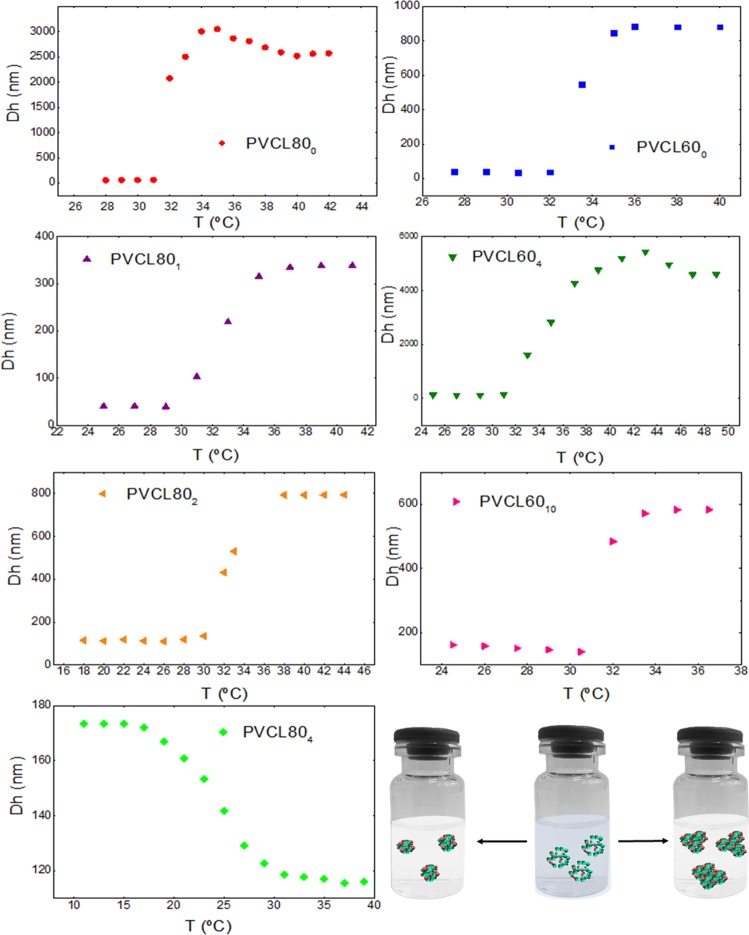
Average hydrodynamic diameters of the synthesized NGs as a function of the temperature of the medium.

The swelling/thermal behaviors of the NGs obtained by DLS are shown in Fig. 4. All the NGs are sized within the nanometric scale when the temperature of the medium is below LCST with a polydispersity index (PDI) values between 0.2 and 0.4. In most of the systems, the response of the NGs to the temperature leads to an increase in average size indicating the aggregation of the particles. However, the PVCL80_4_ NG is the only one which displays collapse without aggregation when increasing the temperature. When considering both set of experiments performed using different initial amount of VCL, a clear trend can be observed: as the BIS concentration increases, the degree of aggregation observed after the Tp decreases (Fig. 4). This behavior could be attributed to stronger intra-molecular interactions present in these experimental conditions.

The average hydrodynamic diameters were determined below Tcp (Table 1). For the same initial VCL concentration, a Dh value increase was observed as a consequence of the increment of the crosslinker amount (BIS). Forcada *et al*.^38,39^ have reported the temperature-sensitive behavior according the nanostructure of gel. Because the fact that the crosslinker BIS reacts faster than VCL, a BIS rich core and a shell of VCL can be formed. According to this, at higher BIS concentration the core size increases resulting in NGs with higher sizes. This behavior can be observed for the two sets of experiment with different initial VCL concentrations. Besides, to the same BIS percentage (see PVCL80_4_ and PVCL60_4_), lower Dh values were obtained for lower initial concentration of VCL. This result could corroborate the presence of a core-shell structure in which the length of the shell decreases with the initial concentration of VCL.

**Table 1:** Average hydrodynamic diameters (Dh), phase transition temperatures (Tp) and cloud point temperatures (Tcp) of the synthesized NGs.

Sample	Dh* (nm)	Tp (°C)	Tcp (°C)
PVCL80_0_	68	31.8	33.3
PVCL80_1_	73	32.5	32.3
PVCL80_2_	148	32.1	32.1
PVCL80_4_	205	24.4	25.2
PVCL60_0_	28	34.5	34.7
PVCL60_4_	142	33.4	33.5
PVCL60_10_	164	31.9	32.6

*Dh measured in the swollen state below the transition phase temperature of the NGs.

Table 1 also shows that Tp decreases with the increase of BIS percentage, in agreement with previous work^30^. As it was mentioned previously, as BIS percentage increases, stronger intra-molecular interactions can be formed, for example hydrogen bonds between the amide groups of the BIS molecules. Accordingly, these hydrogen bonds behave as hydrophobic arrangements in which polymer–polymer interactions prevails on the polymer–water ones^40^. Consequently, the Tp decreases because the polymeric matrix interacts poorly with water molecules.

The phase transition behavior was confirmed by UV-Vis-based turbidity experiments in which the cloud point temperature (Tcp), which represents the phase transition temperature (Tp), can be determined. Transmittance values were recorded at λ = 600 nm against temperature and resulted in a sharp transition between a narrow temperature range, which varied according amount of BIS in the NG, as shown in Table 1. As it can be seen, the values and trends of Tp and Tcp are similar.

### Size and morphology of nanogels

**Figure 5:**
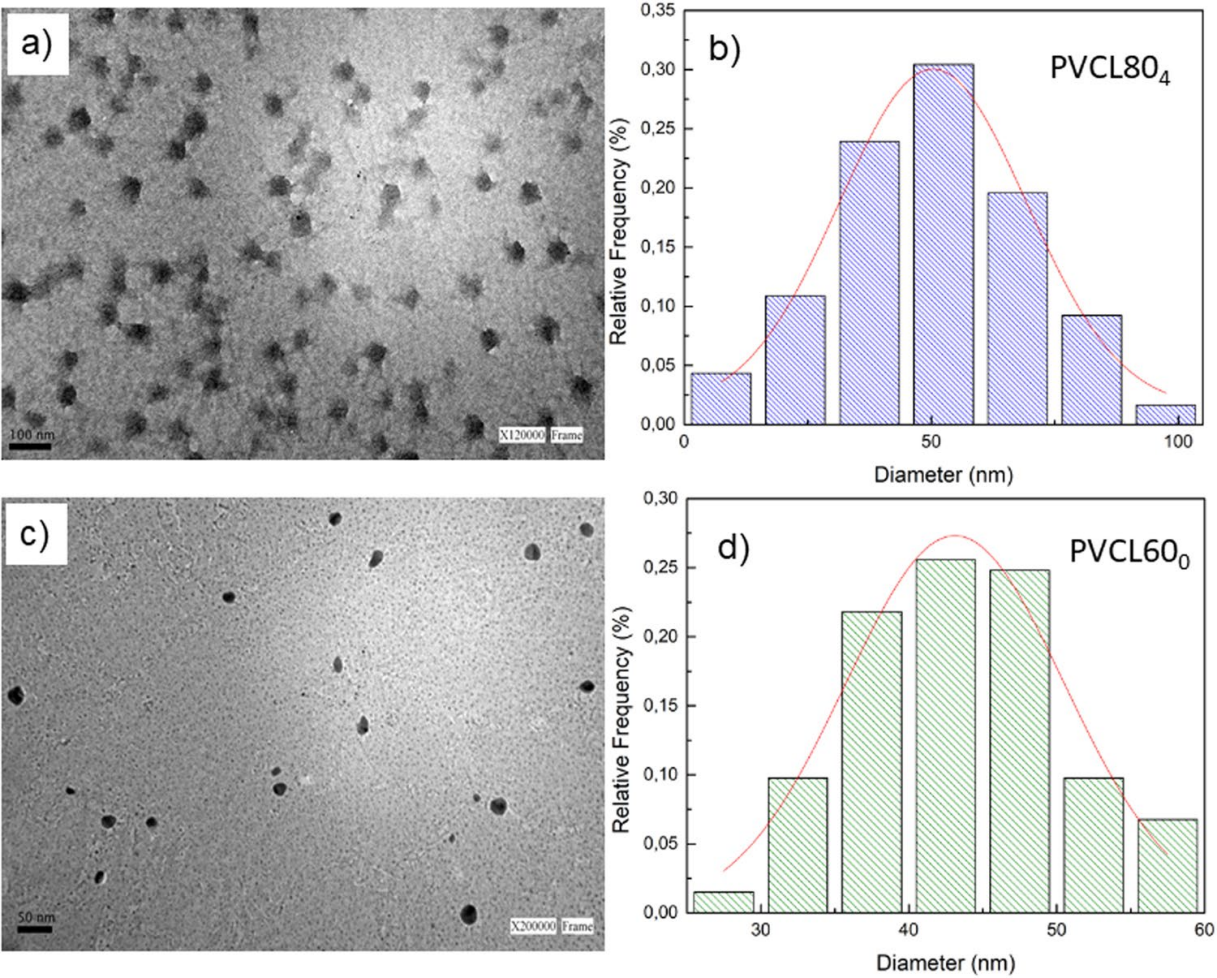
TEM images and size distribution of: PVCL80_4_ (**a**,**b**) and PVCL60_0_ (**c**,**d**).

To examine the size distribution and morphology of the NGs, TEM studies were performed. Figure 5 shows TEM images and size distribution corresponding to PVCL80_4_ and PVCL60_0_.

The TEM images reveal that the size was estimated up to about 40 and 50 nm, respectively, showing low polydispersity in both cases and spherical shape of the particles.

The size values determined by DLS and TEM result similar for the case of the PVCL without BIS (PVCL60_0_). However, the Dh value determined by DLS is higher than the one obtained from TEM for the crosslinked NG PVCL80_4_. This fact can be due to the swelling in solution of the crosslinked NGs networks, this situation did not take place in the case of PVCL60_0_ NGs due to the absence of crossing points and the poor hydrophilic character of VCL.

### Thermogravimetric analysis

**Figure 6:**
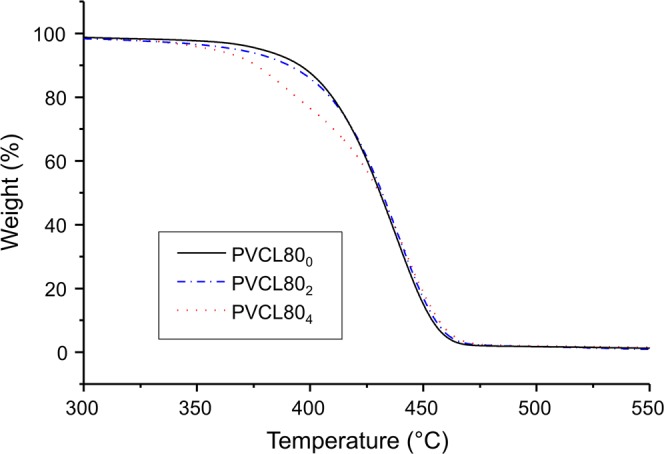
TGA curves of synthetized NGs with different concentration of crosslinker.

Thermal behavior of the dried NGs was qualitatively investigated by thermogravimetric analysis (Fig. 6). In the case of non-crosslinked NG (PVCL80_0_), a single weight loss is observed around 460 °C corresponding to the PVCL decomposition, in accordance with previous reports^41^. For crosslinked nanogels (PVCL80_2_ and PVCL80_4_), thermograms show the beginning of thermal degradation at lower temperatures (around 360 °C) and the major weight loss is similar to the observed for not-crosslinked NG.

### Cytotoxic effect and antiviral properties of NGs

We tested PVCL80_4_ NG as a potential candidate for its use in anti-HIV topical microbicide formulation because it has a collapsed ideal size at physiological temperature. Accordingly, we first evaluated the toxicity of this NG in a cervicovaginal epithelial cell line by using a well-established metabolic activity assay (MTT).

**Figure 7:**
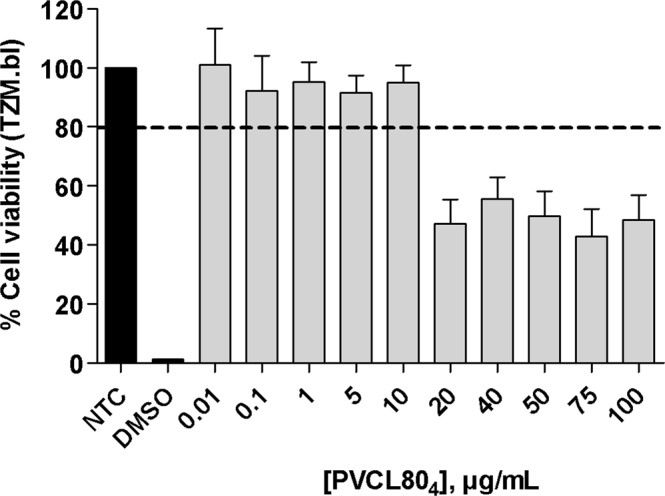
Biocompatibility assays of PVCL80_4_ NG. MTT assay was performed on TZM.bl cells after 48 h of PVCL80_4_ exposure. TZM.bl cells were treated in range concentrations between 0.01 and 100 μg/mL. Medium alone (NTC) and DMSO were used as untreated and cell death controls, respectively. Data is presented as the mean ± SD of three individual experiments performed in triplicate. Abbreviations: NTC = non-treated control; DMSO = dimethyl sulfoxide.

A concentration range of PVCL80_4_ NG (0.01 to 100 µg/mL) was tested for 48 h of exposure. Concentrations were considered non-toxic if the cellular viability was above 80% compared with non-treated control. The results show that PVCL80_4_NG was non-toxic up to 10 µg/mL (Fig. 7). Above 10 μg/mL, a strong increase in toxicity can be observed. This may occur as a result of the increase in the size of the nanogel in the biological environment in which the assay was performed. Probably, this difference in size might be triggering cell death.

**Figure 8:**
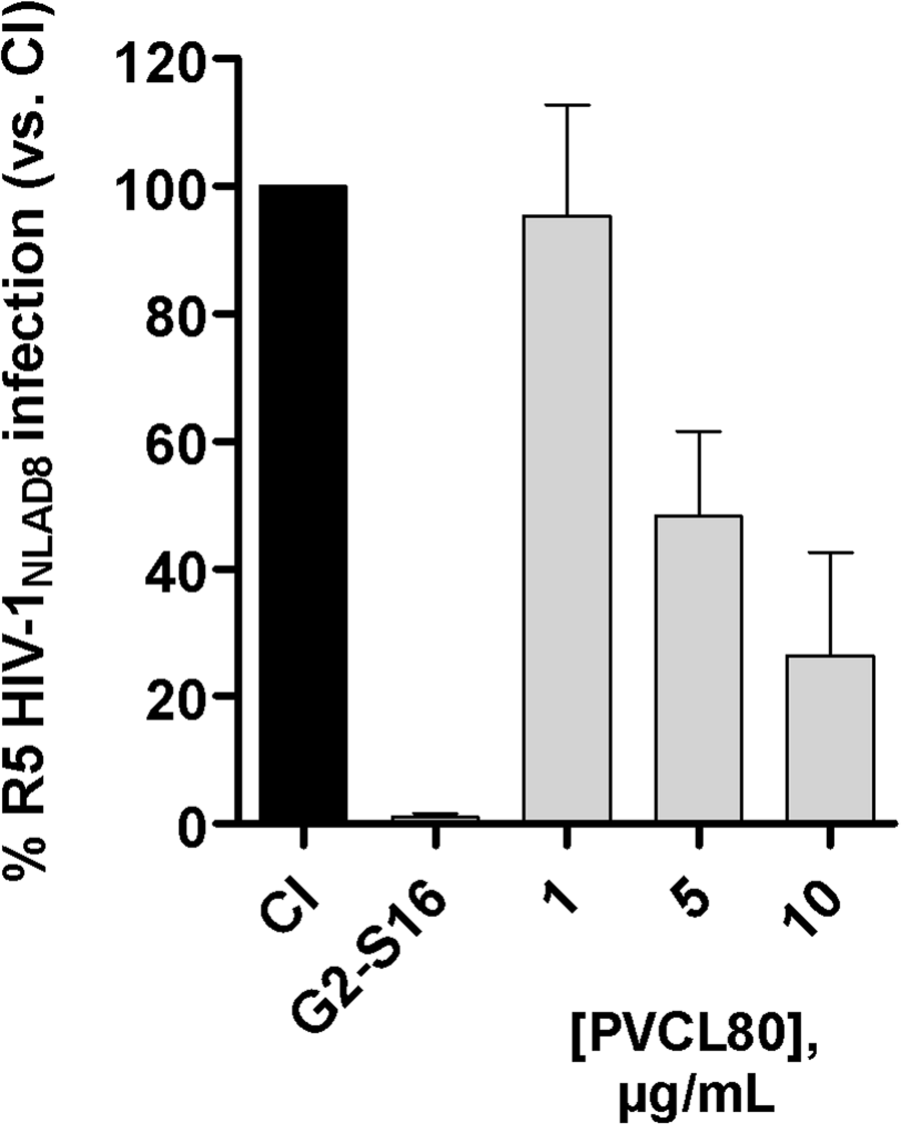
Anti-HIV-1 activity of PVCL80_4_ NG. TZM.bl cells were treated with PVCL80_4_ at several non-toxic concentrations (10 µg/mL, 5 µg/mL and 1 µg/mL) or controls (G2-S16, 10 µM) for 1 h before infection with R5-HIV-1_NLAD8_ isolate (30 ng of p24/10^6^ cells) for 2 h. The percentage of infection was determined at 48 h post-infection by measuring luciferase activity (vs. CI). Data is presented as the mean ± SD of three independent experiments performed in triplicate. Abbreviations: CI = control of infection.

HIV-1 inhibition assays were carried out to determine the anti-HIV-1 efficacy of the PVCL80_4_ NG. We selected the R5-HIV-1_NLAD8_ isolate because it is a widely used laboratory virus predominantly in the first steps of HIV-1 infection^42^. PVCL80_4_ NG showed an inhibitory effect against the R5-HIV-1 isolate *in vitro* since we obtained inhibition rates >70% at the maximum non-toxic concentration (10 µg/mL) (Fig. 8). The ability to inhibit HIV-1 infection decreases as we reduce the concentration of the PVCL NG, exerting a dose-dependent effect.

Synthesized PVCL NGs cannot form anionic moieties which have been demonstrated to be important for antiviral activity^43^, therefore, its inhibitory effect should be due to the functional groups of the polymer matrix. These results support for the first time that PVCL NGs could be considered as candidates for a new HIV-1 microbicide. Nevertheless, more studies are required to reveal the mechanisms involved in this inhibitory effect.

## Conclusions

Thermo-responsive PVCL NGs were easily synthesized by thermo-precipitation in aqueous phase via free radical polymerization. Chemical structure of NGs was confirmed by FT-IR and ^1^H-NMR, the average sizes were determined by DLS and TEM, and thermo-responsive behavior was characterized by DLS and UV-Vis-based turbidity. Most of the NGs get agglomerated with the increase of temperature. However, the NG with initial concentration of 80 mg of VCL and 4% of BIS crosslinking agent (PVCL80_4_ NG) collapses when it is heated. Therefore, this NG has a collapsed nanometric size at physiological temperature which is ideal for biomedical applications. Taking advantage of this thermal behavior, the cytotoxicity and antiviral effect of PVCL80_4_ NG were tested. By using a well-established metabolic activity assay (MTT), it was determined that PVCL80_4_ NG shows no cytotoxic effect at concentrations lower than 10 µg/mL in cervicovaginal epithelial cell line. Finally, we have demonstrated the antiviral effect of PVCL80_4_ NG against the HIV-1 infection by quantification of luciferase activity. To the best of our knowledge, we report the first NG with *in vitro* inhibitory effect against the R5-HIV-1 by itself. Consequently, PVCL NGs could be considered as potential candidates for new HIV-1 microbicide formulations.
